# Transactions of the Pharmaceutical Society

**Published:** 1842-01

**Authors:** 


### Art. XIV.

*Transactions of the Pharmaceutical Society.* Edited by Jacob Bell. Nos. I-VI.—*London,* 1841. 8vo, pp. 293.

Although partaking somewhat of the nature of a journal, from its publication in monthly parts, still, as being the Transactions of a Society, this work comes legitimately within the province of the reviewer. As, however, we purpose treating it as a journal, by occasionally selecting some of its articles for our Third Part, we shall, in the present notice, confine ourselves to the expression of our opinion that the publication is a very valuable one, conceived and conducted in an excellent spirit, and calculated to do much good, not only to the class of readers for whom it is specially designed, but to the professors of medicine in general.

The society from which this publication emanates is of very recent formation, and far from being yet consolidated into the permanent form which it is destined to assume. It has, however, been planned with so much good sense, is carried on with so much zeal, and seems so well calculated to supply a positive want, that we confidently predict its prosperity and usefulness.

The Pharmaceutical Society of Great Britain is to resemble in constitution the colleges of pharmacy now flourishing in various countries of Europe and in America; its main object being to facilitate, promote, and advance the scientific education of young men entering upon that calling, by enforcing their attendance upon lectures on materia medica, pharmacy, chemistry, and botany; and subsequently to grant them a license to practice pharmacy when a competent board of examiners shall deem them fully qualified. It is the avowed intention and purpose of the members of the association to adhere strictly to the dispensing of medicines, and by no means to encroach upon the legitimate functions of medical men. We therefore trust that the general practitioners, who have become to a great extent a mere trading body, will in common justice eventually abandon the practice of retailing drugs in open shops. By so doing they will be able to discharge their professional duties with more credit to themselves and greater advantage to the public. We understand that the chemists are willing to abstain as much as possible from what is termed *counter-practice*, reserving to themselves a privilege which never has been and never can be abrogated, namely, that of stating the properties and doses of the drugs they vend when requested by their customers. It must be admitted that, to prevent people asking and taking their advice is, as things now stand, next to impossible; and for this, the remedy plainly lies at the door of the apothecary. A chemist may sell a medicine-chest along with a volume on the substances it contains, comprising specific directions for the treatment of divers diseases, and of which he may be the author, yet no legal authority can interfere with him for so doing. How then are we to hinder him from proffering his assistance when solicited by some casual visitor suffering from an aching tooth, a cut finger, or a fleeting indigestion? The thing is so palpably absurd, as to be unworthy of argument.

We consider the undertaking as one of a most meritorious and important nature, and wish it every success. It will not only place its own members upon a far better footing as regards the community, but will prove highly beneficial to the profession at large. The sentiments expressed in the subjoined quotation from the fifth number of the Transactions, in a paper written by the editor, are most commendable:

"*The first step* towards an accommodation between apothecaries and druggists is to establish a mutual good feeling and a sense of *professional honour* between them. When this is accomplished, each class will, as a natural consequence, avoid interfering with the duties of the other, in the same manner and for the same reason that physicians abstain from amputating limbs and drawing teeth.

"We have received a letter from a correspondent, complaining of the injury which druggists sustain from apothecaries retailing medicines; and observing that, 'if druggists are prohibited from visiting patients, surgeons ought, at the same time, to be prohibited from keeping retail shops.' As soon as *the first step* is taken, we think these evils will cure themselves. Apothecaries and druggists are not necessarily hostile to each other; they have one common enemy, destructive alike to the interests of both—and that enemy is jealousy. We therefore recommend that they unite their efforts in putting to flight their mutual foe, instead of increasing existing evils ten-fold by constantly attacking each other. We have not done with this subject: the principles which we advocate are those of harmony and fair dealing between professional men, and we shall take every opportunity of exposing the absurdity of party disputes." (p. 228.)

We understand that nearly one thousand of the chemists and druggists of Great Britain have already enrolled themselves as members; and the members are increasing every week.

